# Comparing morbidities of bone graft harvesting from the anterior iliac crest and proximal tibia: a retrospective study

**DOI:** 10.1186/s13018-018-0820-3

**Published:** 2018-05-16

**Authors:** Ying-Cheng Huang, Chun-Yu Chen, Kai-Cheng Lin, Jenn-Huei Renn, Yih-Wen Tarng, Chien-Jen Hsu, Wei-Ning Chang, Shan-Wei Yang

**Affiliations:** Department of Orthopedics, Kaohsiung Veterans General Hospital, No. 386, Ta-Chung 1st Rd, Kaohsiung, 81346 Taiwan, Republic of China

**Keywords:** Bone graft, Iliac crest, Proximal tibia, Visual analogue scale

## Abstract

**Background:**

The anterior iliac crest (AIC) and proximal tibia (PT) are common donor sites for autologous bone graft harvesting. We compared pain levels at these harvest sites on 1 day, 5 days, 2 weeks, 4 weeks, and 8 weeks post-harvest.

**Methods:**

We retrospectively reviewed 18 patients undergoing autologous bone grafting surgery at a level I trauma center between June 2013 and October 2014. Ten grafts were harvested from the AIC group and eight from the PT group. A standard visual analog scale (VAS) was used to rate pain at the harvest sites on postoperative day (POD) 1, 5, 14, 28, and 56 and at the recipient site on POD 1.

**Results:**

There were no statistically significant differences between both groups in age (*p* = 0.474), gender (*p* = 1.00), incidence of harvest site morbidity (*p* = 1.00), and average VAS at the recipient site on POD 1 (*p* = 0.471). VAS at the harvest site on POD 1, 5, and 14 confirmed statistically that pain was more severe in the AIC group than in the PT group (*p* < 0.001). However, no significant difference was observed on POD 28 and 56 between both groups. Pain was significantly less on POD 1 in the PT group at the harvest site than at the recipient site (*p* < 0.001).

**Conclusions:**

The PT is a suitable harvest site, producing statistically less pain for at least two postoperative weeks than the AIC. Besides, patients report less postoperative pain at the PT harvest site than at the recipient site.

## Background

Orthopedic surgeons use bone grafts to augment bone healing, perform arthrodesis, treat nonunions, lengthen bones, or fill defects. Autogenous bone is considered as the best origin for bone grafts, with inherent osteogenic, osteoconductive, and osteoinductive properties [1]. The iliac crest is a commonly harvested site for its abundant cortical and cancellous bone. However, it has been reported that donor site morbidities occur, such as donor site hematoma, infection, incisional hernia, paresthesia, and even fracture [2–4]. Bone grafting at the donor site may result in more severe pain than that from the original surgical procedure [5]. So far, many alternatives to graft harvest sites have been described, such as the proximal tibia (PT), distal radius, distal tibia, and greater trochanter [1]. The advantages of the alternatives include easily accessible source of bone graft, especially if the ipsilateral limb is the recipient site, as well as the theoretically lower complication rates [1, 6–8].

The anterior iliac crest (AIC) and the PT are common sources of bone graft harvesting for surgeries on the ipsilateral lower extremity. Major complication rates at the donor site from 2.4 to 6.2% have been reported in studies of iliac crest graft, resulting in repeat surgery, readmission, prolonged hospital stay, or long-term disability [9–11]. However, major complications associated with the harvest of PT bone graft, including deep hematoma formation, deep infection, joint perforation, donor site fracture, gait disturbance, and nerve injury, have been reported, with an incidence of 0.65 to 2.5% [12, 13].

To the best of our knowledge, there are no comparable studies evaluating donor site morbidities among patients undergoing bone graft harvesting from the AIC or PT. Furthermore, results from reports evaluating harvesting techniques and methodologies for accessing outcomes are conflicting [2, 3, 5, 9, 10]. This study aimed to compare the pain level of autologous bone graft from two different harvest sites on 1 day, 5 days, 2 weeks, 4 weeks, and 8 weeks post-harvest.

## Methods

We retrospectively reviewed 30 patients undergoing lower limb autologous bone graft surgery in a level I trauma center between June 2013 and October 2014. Indications in our study for lower limb autologous bone graft surgery included fracture malunion, nonunion, and bony defect after tumor excision. We included patients whose bone grafts were harvested from either the ipsilateral AIC or the lateral aspect of the ipsilateral PT and who underwent regular postoperative follow-up over 2 years. All surgeries were performed by a single orthopedic surgeon, and all patients were evaluated by an independent observer. Patient exclusion criteria were as follows: (1) age < 20 years, (2) missing pain severity data assessed using visual analogue scale (VAS) during postoperative follow-up, (3) history of bone graft surgery before June 2013, (4) cognition impairment, or (5) paresthesia of the lower limbs. The final study cohort comprised 18 patients. The age, gender, surgical indication, and complications of each patient were recorded entirely. This retrospective study was approved by the Institutional Review Board (IRB) of Kaohsiung General Veterans Hospital (IRB No.:VGHKS17-CT11-04).

Grafts were harvested from the AIC in 10 patients (AIC group) and from the lateral aspect of the PT in eight patients (PT group).

In the AIC group, bone was harvested from the iliac tubercle region approximately 5 cm posterior to the anterior superior iliac spine. A 5-cm skin incision parallel to the iliac crest was made in line from posterior to anterior superior iliac spine to avoid lateral femoral cutaneous nerve injury. Careful dissection with preservation of the tissue planes, elevation of the iliacus muscle, and harvest of inner table graft from the ilium using the curettage technique were performed subsequently to facilitate the closure [1, 9, 14–16].

**Fig. 1:**
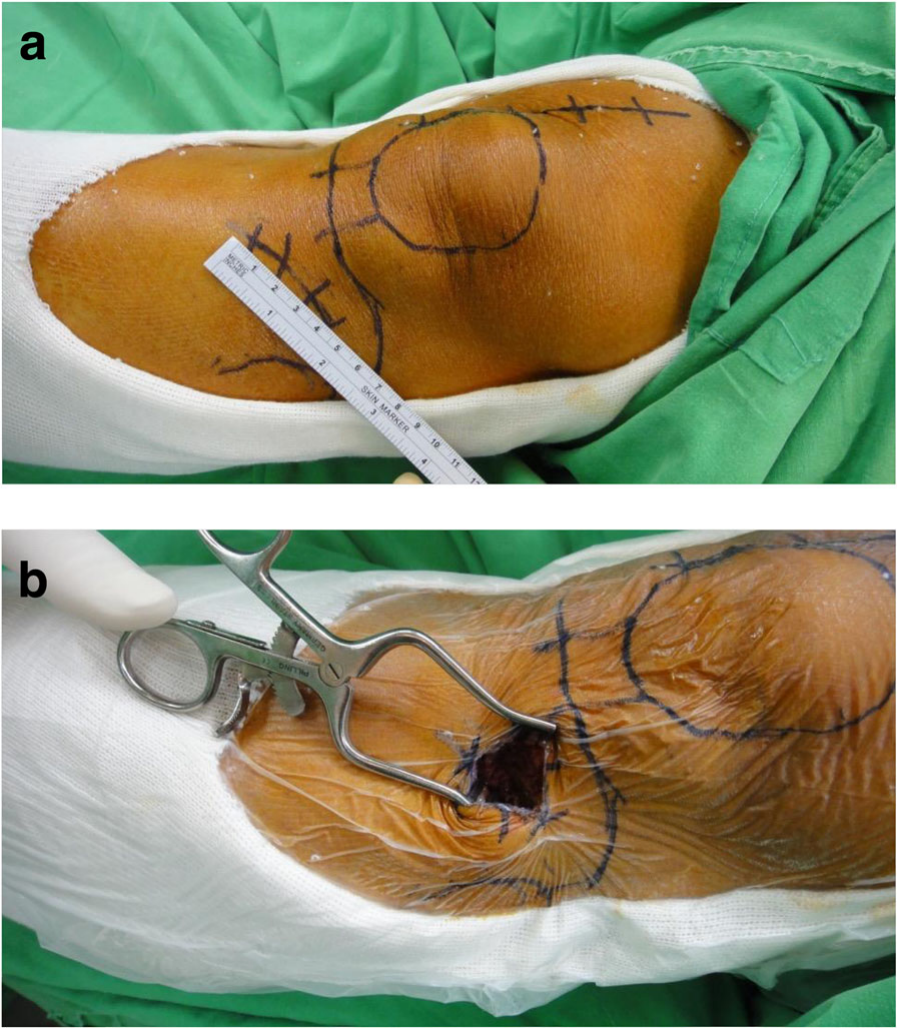
Harvesting the bone graft from the proximal tibia (PT). **a** An incision at the lateral aspect of the PT, just below Gerdy’s tubercle. **b** The entry point into the PT for bone graft harvesting

In the PT group, an incision was made on the lateral aspect of the PT, just below Gerdy’s tubercle, as the entry point into the PT for harvesting (Fig. 1). Careful splitting of the iliotibial band in line with the fascia, followed by osteotomy, was performed to create a window for bone graft harvest using the curettage technique. During this procedure, the knee joint violation was avoided, and the periosteum was repaired during closure [1, 6, 17, 18].

Postoperative treatment during hospitalization in both groups was standardized, including change of dressing with sterilized normal saline, cold packing, and oral analgesics. Analgesics were prescribed for 1 week after discharge. Outpatient follow-up was performed regularly for at least 2 years after the surgery.

The VAS rating was completed by each patient. Patients were required to rate the pain at the harvest sites using a standard VAS on postoperative day 1 (POD 1), 5 days (POD 5), 2 weeks (POD 14), 4 weeks (POD 28), and 8 weeks (POD 56) and at the recipient site on POD 1. The VAS rating ranged from 0 (no pain) to 10 (worst pain ever).

Statistical analysis was conducted using the Statistical Package for the Social Sciences version 22.0 (SPSS Inc., Chicago, USA). Data were analyzed using the Shapiro–Wilk test to determine the normality of the tested variables for the small case number of each group. The Mann–Whitney *U* test was used to compare continuous variables for abnormal distribution in this study. Fisher’s exact test was used to compare the categorical variables. The level of significance was set at *p* < 0.05. Values are represented as mean ± standard deviation.

## Results

There was no significant difference in patient demographic and clinical characteristics between the two groups in age (*p* = 0.474) and gender (*p* = 1.00). The mean ages for the AIC group and the PT group were 49.7 and 47.4 years, respectively.

Moreover, there was no difference between the incidence of harvest site morbidity in the AIC and PT groups (*p* = 1.00). In the AIC group, only one case of harvest site fracture with hematoma was identified. In the PT group, no donor site morbidity was noted. In addition, there were no other donor site complications such as wound infections, deep hematoma formation, tibial plateau fracture, joint perforation, gait disturbance, and nerve injury.

There was no significant difference in the average VAS rating of pain severity at the recipient site on POD 1 between the AIC and PT groups (*p* = 0.471).

**Fig. 2:**
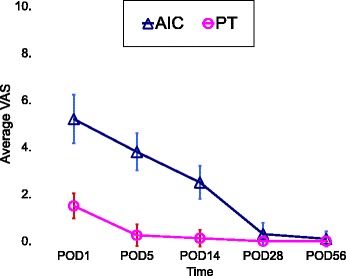
The average VAS rating at the harvest site. The pain was statistically more severe at the AIC site than at the PT site on POD 1, POD 5, and POD 14 (*p* < 0.001), with no significant difference in the VAS ratings on POD 28 and POD 56

The analysis of the VAS ratings on POD 1, POD 5, and POD 14 confirmed that the pain severity at the harvest site in the AIC group statistically outweighed that in the PT group (*p* < 0.001) (Table 1). However, there was no significant difference between the VAS ratings on POD 28 and POD 56 of the AIC and PT groups (Fig. 2).

**Table 1:** Data from anterior iliac crest (AIC) v.s. proximal tibia (PT)

Parameter	AIC	PT	*p* value
Number of patients	10	8	
Average age (years)	49.7	47.4	0.474^a^
Gender (M/F)	6/4	4/4	1.000^b^
Complication rate at harvesting site (%)	*n* = 3 (30%)	*n* = 2 (25%)	1.000^b^
Average VAS of recipient site on POD 1	4.9 ± 0.57	5.25 ± 0.89	0.471^a^
Average VAS at harvesting site			
POD 1	5.2 ± 1.0	1.5 ± 0.5	< 0.001^a^
POD 5	3.8 ± 0.8	0.3 ± 0.5	< 0.001^a^
POD 14	2.5 ± 0.7	0.1 ± 0.4	< 0.001^a^
POD 28	0.3 ± 0.5	0	0.216^a^
POD 56	0.1 ± 0.3	0	1.000^a^

Data are expressed as mean ± standard deviation

Abbreviations: *VAS* visual analog scale, *POD* postoperative day

^a^Mann–Whitney *U* test

^b^Fisher’s exact test

In addition, the pain severity between the recipient site and harvest site was compared in our study. In the AIC group, there was no significant difference in the average VAS rating on POD 1 between the recipient site (4.9 ± 0.6) and harvest site (5.2 ± 1.0) (*p* = 0.598); however, in the PT group, the average VAS rating on POD 1 for the harvest site (1.5 ± 0.5) was significantly lesser than that at the recipient site (5.3 ± 0.9) (*p* < 0.001) (Table 2).

**Table 2:** Comparison of the VAS on postoperative day 1

	Recipient site	Harvesting site	*p* value
AIC	4.9 ± 0.6	5.2 ± 1.0	0.598^a^
PT	5.3 ± 0.9	1.5 ± 0.5	< 0.001^a^

Data are expressed as mean ± standard deviation

Abbreviations: *VAS* visual analog scale, *POD* postoperative day, *AIC* anterior iliac crest, *PT* proximal tibia

^a^Mann–Whitney *U* test

## Discussion

The most common donor sites for harvesting include the iliac crest, greater trochanter, and PT. The choice of bone graft harvest site depends on the surgeons’ preference and the patient’s injured limb. However, different techniques have led to various complications and morbidity associated with the donor site in the past, necessitating the evaluation of the overall efficacy of the procedure. We must account for the quantity, quality, and morbidity of different harvest sites, especially regarding pain from the patient’s perspective.

Mauffrey et al. conducted a prospective in vivo quantification study that indicated no significant difference in the volumetric amount of cancellous bone available for harvest between the PT site and the AIC site [5]. Chiodo et al. reported histologic differences, such as osteogenic and hematopoietic progenitor cell content between the iliac bone and the tibia shaft, and that the former was superior to the latter [19]. However, Takemoto et al. reported no significant difference in the mRNA levels of all types of bone morphogenetic proteins from different harvest sites, which implies that the choice of donor site should not depend on the inherent differences in the osteoinductive and osteogenetic potential of the bony material itself [20].

Although the iliac crest is the most common donor site for autogenous cancellous bone graft, it has associated donor site morbidity as high as 7% [4, 10, 21]. In our study, regarding the incidence of harvest site morbidity, there was only one case of donor site fracture in the AIC group. Alt et al. claimed that a sufficient amount of cancellous bone can be harvested from the proximal tibial metaphysis and that the risk of postoperative fracture is not increased [22]. In our study, no harvest site morbidity was identified in the PT group.

Techniques for iliac cortex harvesting offer improvement in cosmetics and donor site pain [23]. A retrospective review that compared the morbidity associated with anterior and posterior iliac crest harvest sites found that both sites have an overall complication rate lower than those previously reported and that a posterior site has a substantially lower risk of overall postoperative complications than an anterior site (2 vs. 23%) [4]. Mauffrey et al. also assessed the response to each graft site from the patient’s perspective and reported that the pain experienced by patients after AIC grafts was higher than that at the PT or olecranon [5]. In our study, we had a similar result with the evaluation of VAS ratings on POD 1, POD 5, and POD 14, which indicated that the pain at the PT harvest site was statistically less severe than that at the AIC. Nevertheless, as the patients recovered, there was no significant difference in pain severity 1 month postoperatively between the two groups (Fig. 2).

Baumhauer et al. compared patient-reported outcomes of acute and persistent pain at 1 year after foot and ankle surgery, in which bone graft harvest site pain at 3 weeks showed more significant pain in the iliac crest than proximal tibia and calcaneus, respectively [24]. In our study, there was no significant difference between the VAS ratings on POD 28 and POD 56 of the AIC and PT groups, and only one case of harvest site fracture with hematoma was identified in the AIC group. On POD 1, there was no significant difference in the pain level at the recipient site between the two groups. This finding suggested that the postoperative pain severity in both groups was equal, but the pain was more severe with iliac crest graft harvests at early time points (POD 1, 5, and 14), although differences resolved by 4 weeks after surgery. More importantly, PT harvesting gave rise to not only less pain at the donor site for at least two postoperative weeks but also significantly less pain than that at the recipient site on POD 1 (Table 2).

The issue about the bone graft volume was ever evaluated by the syringe according to certain literatures. In our study, we harvested the bone graft as much as possible during surgery. Even the graft volume from proximal tibia was larger than the iliac crest, the proximal tibia donor site gave less complication than the iliac crest, according to the report of Salawu et al. [25], and the present study’s result showed there was less pain in the proximal tibia harvest site. The lack of comparison of quantity of harvest graft is one our limitations, and bone harvest at two sites of the same person simultaneously for the comparison of volume was also not practicable in our retrospective study. What we took into account was the comparison of pain severity between the anterior iliac crest and proximal tibia harvest sites, and the result showed less postoperative pain at the proximal harvest site for at least two postoperative weeks than at the anterior iliac crest. Besides, the pain severity of PT harvest site is less than the recipient site.

Limitations in our study included the retrospective study design, the limited sample size for each group, comparative analysis of the quantity of graft, the detailed consumption of analgesics, functional scale, clinical outcomes, and the bias of the patient’s subjective tolerance to pain. In addition, there was no evaluation of graft volume and quality or of the mechanical factors resulting from the implant choice for surgery that may influence the VAS rating at the recipient site and bone union.

## Conclusions

To summarize, the PT and AIC are optimal donor sites for autologous bone grafting. However, harvesting from the PT decreased pain at the donor site for at least two postoperative weeks. In addition, patients appeared to experience lesser pain at the PT donor site than at the recipient site on postoperative day 1.

## Abbreviations

AIC: Anterior iliac crest
POD: Postoperative day
PT: Proximal tibia
VAS: Visual analog scale
